# A Novel Blockchain Framework for Industrial IoT Edge Computing

**DOI:** 10.3390/s20072061

**Published:** 2020-04-07

**Authors:** Xuesong Xu, Zhi Zeng, Shengjie Yang, Hongyan Shao

**Affiliations:** 1 Key Laboratory of Hunan Province for New Retail Virtual Reality Technology, Hunan University of Technology and Business, Changsha 410205, China; zrh1407@126.com (Z.Z.); 2633@hutb.edu.cn (S.Y.); dannyxu@hnuc.edu.cn (H.S.); 2 Hunan Province Engineering Research Center of Ecologic Environment Big Data and Intelligent Decision-Making Technology, Changsha 410205, China

**Keywords:** blockchain, industrial internet of things, edge computing, lightweight

## Abstract

With the rapid development of industrial internet of thing (IIoT), the distributed topology of IIoT and resource constraints of edge computing conduct new challenges to traditional data storage, transmission, and security protection. A distributed trust and allocated ledger of blockchain technology are suitable for the distributed IIoT, which also becomes an effective method for edge computing applications. This paper proposes a resource constrained Layered Lightweight Blockchain Framework (LLBF) and implementation mechanism. The framework consists of a resource constrained layer (RCL) and a resource extended layer (REL) blockchain used in IIoT. We redesign the block structure and size to suit to IIoT edge computing devices. A lightweight consensus algorithm and a dynamic trust right algorithm is developed to improve the throughput of blockchain and reduce the number of transactions validated in new blocks respectively. Through a high throughput management to guarantee the transaction load balance of blockchain. Finally, we conducted kinds of blockchain simulation and performance experiments, the outcome indicated that the method have a good performance in IIoT edge application.

## 1. Introduction

Blockchain technology integrates encryption, peer-to-peer transmission, consensus, distributed storage and other technologies. Its potential application value has become a hot topic of discussion, attracting a wide range of research and development interests in Industrial Internet of Things (IIoT). IIoT applies to a large number of edge sensor devices with limited storage, computing, and bandwidth. There may be hundreds of data sources and data requirements in the field device layer of the industrial internet. The distributed and massive data traffic become bottlenecks to respond to the required quality of service (QoS). Data acquisition and exchange are the base of the system operation. How to reduce the delay of data exchange to achieve effective data exchange is a fantastic challenge. Blockchain technology [1] as the core technology of digital currency [2], such as Bitcoin and Ethereum, can solve the problem of trust-building among nodes of decentralized system through the verification and consensus mechanism of distributed nodes [3], thus completing the transfer of value while transmitting information, and realizing the significant transformation of current network architecture from “information Internet” to “value Internet”. The transaction privacy, security [4,5], data invariance and auditability [6,7,8], integrity and fault tolerance of blockchain make them widely used in many fields besides cryptographic, including identity management [9], intelligent transportation [10,11,12,13,14], supply chain finance [15], Internet of Things [16,17], etc. Lu and Liu [18] use blockchain encryption technology and an anonymous trusted transaction mechanism to improve decentralized control of industrial Internet applications. Li et al. [19] introduce blockchain technology to better reduce the operating cost of distributed energy management and resist threats and attacks. Dai et al. [20] investigate integrating blockchain with IoT and name synthesis of blockchain and IoT as BCoT, then provide a comprehensive survey on BCoT. The traditional IIoT systems are vulnerable to malicious attacks and single point of failure due to the centralized architecture, which become a new sensing paradigm owing to its merits such as cost effectiveness, mobility and scalability. Huang and Dai et al. [21] propose a blockchain-based MCS system (BMCS) and exploit miners to verify the sensory data and design a dynamic reward ranking incentive mechanism to mitigate the imbalance of multiple sensing tasks. Wang et al. [22] design a highly-efficient distributed tensor-train decomposition method for IIoT Big Data. Suárez-Albela et al. [23] introduces blockchain technology to better reduce the operating cost of distributed energy management and resist threats and attacks. 

IIoT usually contains resource constrained edge computing devices. Low computing power, low memory, and low battery capacity is the characteristics of an IIoT edge computing. Therefore, the system needs a lightweight algorithm, which should balance computing and resource consumption. IIoT lacks effective data sharing mechanism, which makes it difficult to realize the valuable data interconnection. 

The novel contribution of this paper is to propose a Lightweight Blockchain Framework (LLBF) for IIoT and its edge application. Firstly, according to IEC62443 standard, we design a framework includes two scalable virtual networks: a resource constrained layer (RCL) and a resource extended layer (REL). The corresponding blockchain operations are designed for edge computing units with different resource capabilities in the layered region. In RCL, resource constrained nodes are clustered into cluster header as an edge computing unit. Only the cluster head node is responsible for managing blockchains. REL includes nodes with certain computing power to collaboratively manage common blockchains. Secondly, we redesign the structure and size of the block. In RCL layer, the block policy head has four parameters. Block body only stores basic control and state information. We initialization maximum block body size is 256 Kb in the RCL edge computing units, and the total cache size of each device block is 2 M. If the block continues to increase, the block will be synchronized to the REL layer with greater processing capacity. Finally, we incorporate a number of optimizations which include a lightweight consensus algorithm, a trust right confirmation algorithm, and a high throughput management mechanism to separate the transaction traffic from the data flow. All this optimization is in order to improve the throughput of blockchain and ensure it is self-scaling and adjust the transaction load balance.

The remainder of this paper is summarized as following. Section 2 presents an overview of related work and describes problem of IIoT. In Section 3, we propose a layered blockchain model and RCL, REL schemes. A consensus algorithm, trust right mechanism, and high-throughput management algorithms are carried out respectively. In Section 4, a realization of the blockchain transaction case in industrial internet is presented. In Section 5, we designed a numerical simulation and performance evaluation. Finally, we conclude our work in Section 6.

## 2. Related Works

Several issues relating to this study are reviewed in this section. Foremost, issues of blockchain-based IIoT technology analysis, and academic literature about layered industrial internet architecture research, are addressed respectively.

### 2.1. Blockchain-Based IIoT Technology Analysis 

Blockchain system uses encryption technology to protect data and relies on strong consistent algorithm to resist external attacks. Merkle tree and its commonly used variants in blockchain systems support simplified payment verification and improve the efficiency of blockchain operation, which makes it possible to use blockchain technology on industrial internet devices. IIoT mainly applies instrumentation, sensors, and edge devices to machinery, vehicles in the transport, and the energy and industrial sectors. Combined with blockchain technology, industrial control system can benefit from low operating cost, decentralized resource management, robustness against threats and attacks, etc. By allocating computing and storage requirements among all devices in the network, blockchain establishes a peer-to-peer network, which reduces the installation and maintenance costs of centralized cloud, data center and network devices. This communication mode addresses the issue of single point failure. Blockchain solves the privacy problem of Internet of Things by using encryption algorithm. It also resolves the reliability problem in the IoT through the use of tamper proof ledgers [24]. However, numerous academic literatures and some research on blockchain-based IIoT technology has only focused on general applications of the blockchain [25,26,27]. They have no insight into the blockchain challenges associated with the Internet of Things. Arias et al. [28] emphasize various security, privacy and performance problems of blockchain application in the field of IoT. Nevertheless, IoT has different multi-layer network architecture from the IIoT. Makhdoom et al. [29] put forward a lightweight architecture of the smart home and proposes a way to reduce computing density and transmission delay solutions to problems such as lateness and scalability. The effect of its practical application is not considered in depth. Dorri et al. [30] propose a scheme for configuring and managing IoT devices using blockchain smart contracts. Solve the security and synchronization problems in the terminal server model. In the IIoT environment, network nodes do not necessarily trust each other. Encryption hash link and distributed consistency mechanism ensure that the data stored on the immutable blockchain will not be changed or deleted, but the blockchain mechanism does not guarantee the reliability of the data in the source. Wang et al. [31] puts forward a cloud-edge computing framework for Cyber-Physical-Social Services. Therefore, some recent researches focus on integrating trust mechanism and performance optimization into blockchain-based IIoT applications.

However, due to the inherent characteristics of industrial internet, such as lack of central control, heterogeneous devices and limited computing capacity, traditional blockchain technology and data security protection have challenges, such as:

(1) Resources constraints: Industrial internet consists of numerous terminal devices, which need to communicate with each other in time. However, most devices have limited resources, including bandwidth, computing and memory. This requires increasing many blocks containing a large number of transactions to the blockchain every second, which requires a consistent method with low latency [29].

(2) Multi-center management: The traditional industrial internet relies on a hierarchical agent communication model, in which all devices are identified, verified and connected through the central control system. Because of the large-scale devices, the centralized server is sometimes difficult to meet the real-time interaction needs of decentralized devices [30]. In general, IIoT has some constraint in performance requirements, and prepare to save a certain degree of data integrity in calculation and energy consumption. One way to achieve this is to relax the proof of work to reduce the calculation requirements.

(3) Data security: The key parameters and sensitive data in industrial internet put forward higher requirements for data privacy and security protection. The heterogeneous network structure needs to ensure the data security protection from the sensor, communication protocol and the whole process of information processing [31,32]. 

Although some scholars have proposed a distributed access control method to confidential information, it brings too much delay and overhead. IPSec and TLS are used in reference [32] to provide authentication and privacy, but they are not suitable for various computing devices with limited resources due to the large computing cost. Their consensus mechanism and block validation are contrary to the requirements of complex blockchain solutions.

IIoT is a system of physical objects that can be monitored, controlled by edge devices to enable ubiquitous computing services. Unfortunately, based on the limitation of memory, power and computational resources, they always delegate industrial internet application tasks to cloud computing in management information layer. In industrial internet, the computing resources needed to participate in block consensus are heterogeneous, distributed and low-power, which limits the number of blocks that can be mined by blockchain nodes. Nodes in heterogeneous networks have an obligation to agree on the correctness and timing of transactions contained in newly excavated blocks. Otherwise, copies of blockchains of nodes may be inconsistent, leading to bifurcation of blockchains. Therefore, adding one new block to the industrial blockchain needs to solve the problems of high computational requirements.

### 2.2. Layered Industrial Internet Architectures Research

With the continuous growing interest in the blockchain and IIoT, many new integrated industrial internet platforms and systems have been proposed in the academic studies to provide efficiency and security solutions [33,34,35,36,37]. The core of IIoT is a data-driven network built on the Internet of Things and traditional internet network. Data, storage, share, and security is the common foundation of industry internet. Through ubiquitous connectivity, the industry internet has the capacity to collect data from various factors of production, such as equipment, software, personnel and systems [33]. 

**Figure 1:**
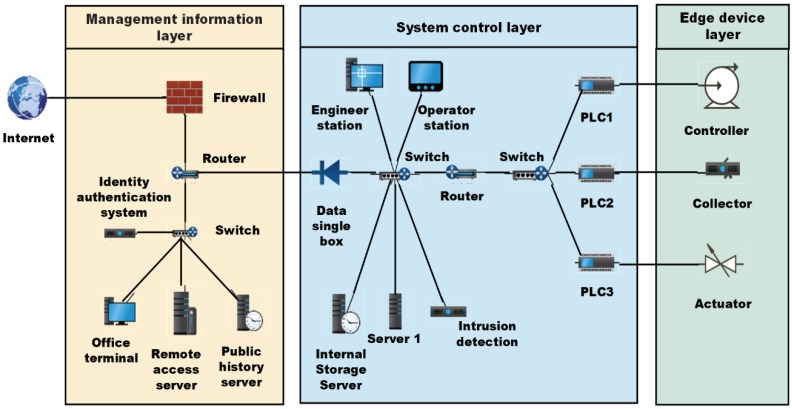
Typical architecture of layered industrial internet system.

Due to the lack of standardization of industrial internet at present, different literature discusses industrial internet architecture and its tasks slightly differently. Damiano, et al. [36] describe Blockchain 3.0 development and all the properties obtained by the blockchain trustless decentralization to other systems which are built on top of blockchain technology. In Zou et al. [37], a five-layer network architecture is proposed, including a sensing layer, abstract layer, service management layer, application layer and business layer. Qing et al. [38] propose a three-layer architecture model including sensor, network and application layer is introduced. Mary, et al. [39] consider the differences between the terminal and the sensing layer, propose a four-layer network architecture. The differences of these network structures are of great importance, which affect the security and privacy protection policies of industrial internet system. Because of this diversity, it is difficult to develop a standard security protocol for all types of industrial internet devices and application systems [40]. According to IEC62443 standards [23], we can divide industrial internet as edge device layer, system control layer and management information layer, whose functions include computing, communication, sensing and driving functions. Through cloud-edge-end services, massive data storage, management and computation are clearly understood in this network architecture. The architecture is illustrated in Figure 1. Take into account this network architecture, RCL and REL are defined respectively. 

(1) Edge device layer: Edge device layer includes low-power devices or embedded platforms such as sensors, collectors, controllers and execution units. The device in this layer is identified as a node with limited processing and storage capacity, which is called a Class 0 device and belongs to RCL. The energy consumption of the device is an important consideration.

(2) System control layer: The layer is responsible for collecting data from the edge device, which can be comprised of a gateway, central controller, data collector, etc., for handling inbound and outbound communication with terminal device layer. In addition, data from the field device layer is processed in this layer to meet the real-time requirements of the application. In terms of computing and storage capacity, the devices in this layer are more powerful than those in the terminal device layer, which are called Class 1 devices and belong to the REL.

(3) Management information layer: Management information layer is responsible for information processing, storage and visualization functions and belongs to the core resource layer. In this level, Management Information Systems (MISs) use all of their resources and services to support applications [41]. The data aggregated by the edge device layer will be received and stored in the cloud blockchain storage. An MIS also provides intelligent services to manage and control RCL or REL data. The data can be stored in a cloud database or in RCL and REL blockchains.

As a result, the integration structure brings a comprehensive security framework with enhanced privacy preservation, data ownership and secure data sharing. It also eliminates totally single point failure bottlenecks, prevents efficiently disruption of centralized services, and enhances significantly data availability.

### 2.3. Summary

IIoT are physical object systems that can be monitored. Unfortunately, due to the lack of memory, power consumption and computing resources of edge devices, they always entrust industrial internet application tasks to cloud computing in the management information layer. In the industrial internet blockchain, the computing resources needed to participate in the block consensus are heterogeneous, distributed and low-power, which limits the number of blocks that can be mined by blockchain nodes. Nodes in heterogeneous networks are required to agree on the correctness and timing of transactions contained in new mining blocks. Otherwise, block copies of nodes may be inconsistent, leading to blockchain fork. Therefore, adding new blocks to the industrial blockchain needs to solve the problem of high requirements of traditional consensus computing. Blockchain structure is a series of data blocks linked together by hash values. Cryptographic hash links and distributed consensus mechanisms ensure that the data stored on an immutable blockchain can’t be altered or deleted. However, blockchain mechanisms do not guarantee the trustworthiness of data at the origin. More recent works focus on integrating the trust mechanisms and performance optimization into blockchain-based industrial internet applications [42].

## 3. Blockchain Framework Structure and Design

### 3.1. A Layered Lightweight Blockchain Framework

**Figure 2:**
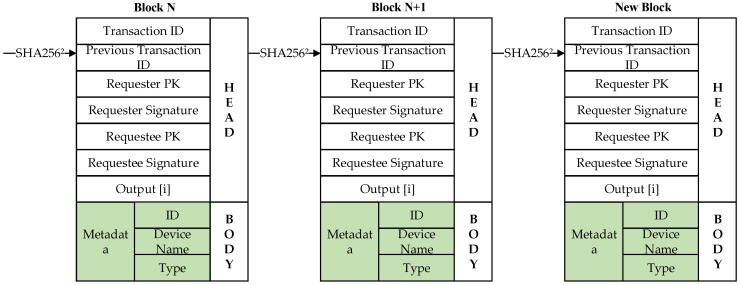
Transaction data structure.

According to the definition in the previous section, a Layered Lightweight Blockchain Framework (LLBF) will be designed. The nodes in the LLBF are composed of various edge computing devices, including execution units, centralized controllers, network devices and servers. In order to ensure the scalability of blockchains and reduce network delay, edge devices or edge computing units in RCL are grouped by functional attribute clustering, and each cluster selects cluster head (CH) to manage the corresponding blockchain. If there is excessive delay of some nodes in industrial internet, then these nodes can be re-clustered and change their clusters. Generally, CH nodes that remain online for a long time are selected and the basic tasks in the cluster are stable. Therefore, the RCL blockchain is not affected by the dynamic changes of devices. Asymmetric encryption, digital signature and cryptographic hash functions, such as SHA256, are utilized to protect all kinds of transactions generated by nodes. A transaction structure of block data in LLBF is shown in Figure 2, which contains seven attribute fields and one data field. The first field records the current transaction ID, and the second field is the aforementioned transaction pointer, which is linked into blocks through pointers. The next four fields are the PK and digital signature. The digital signature belongs to the requester and requestee. The seventh field is the Output [i], i = 0,1,2 set of the requester. Output [0] denotes the number of accepted transactions generated by requester, Output [1] denotes the number of transactions rejected by requested, and Output [2] is the PK hash used in next transaction of requester. The final field is “metadata”, which provides a record of the operations required by the device node, including ID, device name, and operation type.

Each CH decides independently whether to retain or discard a new block based on the communication received from the transaction participant (including the requester and the requested), which may result in different versions of blocks in each CH. Corresponding to the accounting operation of each cluster center node, the blockchain model of this layer does not need to coordinate the block consistency in real time, thus reducing the block synchronization overhead. Data resources are summarized through the REL layer, so the resource extended nodes in the REL verify the block consistency within a specified waiting period, thus addressing the problem of insufficient computing performance of equipment resources in RCL layer. 

**Figure 3:**
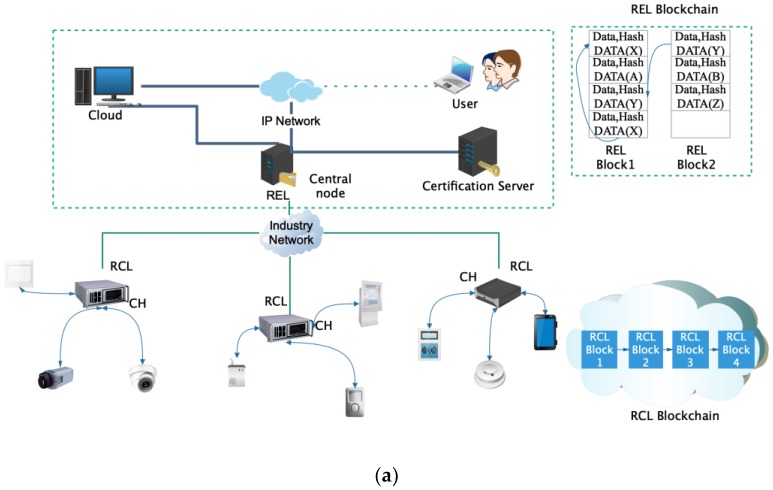

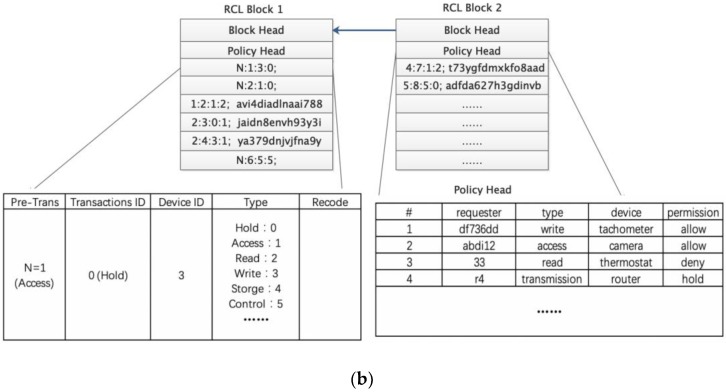
A Layered Lightweight Blockchain Framework (LLBF): (**a**) Layered Blockchain network model; (**b**) Structural design of resource constrained layer (RCL) block.

Figure 3a describes the network structure model of layered-blockchain, and Figure 3b presents the design of blocks of RCL layer.

Each RCL block contains a block head and a policy head. The block head stores the previous block hash, and the policy head maintains an access control lists. These control lists define RCL transactions and rules for communicating with REL. The policy head has four parameters. The first parameter is the network device ID of the requester transaction. The second parameter represents the request requirement, including data writing, data reading, access control, monitoring and data transmission. The third parameter is the specified target device, and the fourth parameter is the operation permission. All block structures are indicated on the left side of the figure, identifying the specific content of a transaction, including the current transaction, transaction chain, transaction type, access device and related operation record information. Each REL block contains a block head and a transaction body. The block head stores the previous block hash value, generator ID, and verifier’s signature. If illegitimate access attempts to change previously transactions, the hash value of the corresponding block will remain on the block and there will be inconsistencies, thus exposing this attack. In the RCL layer, because of the equipment resources constraints, the CH block head only stores hash value, the block body only stores basic control and state information. The initialization maximum block body size is 256 Kb, and the total cache size of each device block is 2 M. If the block continues to increase, the block will be synchronized to the REL layer with greater processing capacity

### 3.2. Improved Consensus Algorithm

In LLBF, a time consistency algorithm is therefore proposed to replace traditional resource intensive algorithm. The block generator needs to be randomly selected in this algorithm. Each block CH must wait for a random period time T before generating a new block. In this case, the CH must delete these transactions from its block and request other CH to wait for a certain interval time to achieve synchronization. The maximum interval time is double delay time of end-to-end device. The default maximum for the consensus period is 5 min (about 1/2 of the Bitcoin mining cycle). The minimum time of consensus cycle is about twice of the maximum end-to-end delay between nodes, which provide enough time to propagate new blocks generated by other cluster centers. When the number of blocks exceeds the threshold (perform according to the network environment and performance requirements), CH will discard the blocks generated by their neighbors. Each CH node in the blockchain must verify the new block received from other nodes, and then attach it to the chain. To verify the block, CH first verifies the signature of the block generator. Consensus algorithm 1 outlines the process of verifying a block consensus.

As showed in the block data structure in Figure 2, the link between consecutive transactions is established by the requester’s current transaction and the hash of the previous transaction PK. CH compares the hash of the requester’s PK in (X) with the aforementioned transaction output [2]. If the requester agrees to the transaction, output [0] will increase 1. Otherwise, the output [1] is increased by 1. CH only checks successful transactions output [0] of (X) number or rejected transactions output [1] number in transaction verification. After that, the requested signature is verified using its PK in X. The detail describes the implementation process shown as bellowing consensus Algorithm 1.
**Consensus Algorithm 1**Input: Transaction(X)Output: True(T) or False(F)Requester verification:1. if (hash((X.requester) = X(output[2]) then2.   return F3. else4.   if (X.requester-PK redeem X.requester-Signature) then5.     return F6.   end if7. end ifOutput verification:8. if(X.output[0] − (X−1).output[0]) + (X.output[1])-(X−1).output[1]>1) then9.    return F10. end if  Requestee verification:11. if (X.requestee-PK redeem X.requestee-Sign) then12.    return T13.  end if14. end if

### 3.3. Dynamic Trust Confirmation Mechanism

In the IIoT, the number of device changes will be quite remarkable, which conducts to verify all transactions and blocks will be costly. To solve this problem, RCLBC uses dynamic trust confirmation mechanism to build mutual trust in CH nodes of the cluster center. This mechanism introduces absolute trust and relative trust models to gradually reduces the number of transactions for confirmation in each new block. 

(1) Absolute trust: If the cluster center node CH1 has previously verified at least one of the blocks generated by cluster center CH2, then it has unequivocal evidence of CH2. 

(2) Relative trust: If CH1 does not have direct evidence of CH2, but CH2 and a cluster center CHX trusted by CH1 are found to be valid, then CH1 has unintended evidence of CH2. Therefore, each cluster center maintains a dynamic list of record relevant information to establish a trust relationship model, as showed in Table 1. Cluster center decreases the trust rating of other non-conforming nodes through trusting mechanism. The core idea of the dynamic trust right confirmation mechanism of network is that the stronger the trust of nodes collecting other nodes to generate new blocks, the lower the verification cost in this block, so as to improve the consensus and right confirmation efficiency of blockchain.

### 3.4. Dynamic High-Throughput Management

In LLBF, a dynamic high throughput management (DHM) mechanism is proposed, which actively monitors blockchain throughput and network utilization. At the end of a consensus period, each CH calculates utilization as the ratio of the total number of new transactions generated to the total number of transactions added to the blockchain. Assuming that the network has N nodes, Ct is the consensus cycle, M is the number of CH after clustering and S is the average speed of transactions generated by nodes per second, the utilization ratio θ of blockchain network is expressed as follows:

(1) $$\theta =\frac{N\times S\times C_{t}}{M\times T_{\max}}$$

The above equation shows that there are two ways to adjust utilization ratio: 

(1) Changing consensus period Time (T). This value T will be determined according to the corresponding industrial network delay and blockchain block generation frequency; 

(2) Changing M. Since each CH can generate a block during the consensus period, there will be more overhead if the clustering state of nodes is readjusted. 

Therefore, if θ exceeding $\theta_{max}$, the first mode is used. DHM checks whether consensus cycles can be reduced and ensures that θ is in the median attachment of the expected range. If the consensus cycle can’t be reduced, the cluster relationship between CH and nodes can be adjusted by re-clustering to achieve the extension of LLBF. We reset the consensus cycle to the default value, otherwise it will remain unchanged at the minimum threshold, so if the utilization rate increases above its threshold, the network reconfiguration will be started, in which the increase in the number of participating nodes can provide higher throughput. When the utilization rate drops below $\theta_{min}$, the reverse operation is adopted. According to the above operations, the dynamic management of blockchain flux under different network states can be realized. We describe the DHM Algorithm 2 as below.
**DHM Algorithm 2**Input: θ1. if (θ > $\theta_{max}$) then2.   calculate *Ct-new* based equation1, while θ = ($\theta_{max}$ + $\theta_{min}$)/23.   if (*Ct-min <= Ct-new*) then4.     Replace *Ct* use *Ct-new*5.   else6.     reset *Ct* as default value7.    end if8.  if (θ < $\theta_{min}$ then9.   calculate *Ct*-new based equation1, while θ = ($\theta_{max}$ + $\theta_{min}$)/210.   if (*Ct-new<= Ct-max*) then11.     Replace *Ct* use *Ct-new*12.   else13.     reset *Ct* as default value14.    end if15. end if

The percentage of transactions to validate also depends on the number of CH nodes N. As the number of CH validators increases, the percentage of transactions that each validator node needs to validate decreases. As a result, there is a risk that invalid transactions will not be detected in the given block. The probability P (not detection invalid transactions) can be calculated as follows:

(2) $$P(\mathrm{not}\;\mathrm{detection}\;\mathrm{invalid}\;\mathrm{in}\;T_{\mathrm{inval}})={\left(\frac{T_{\mathrm{total}}-T_{\mathrm{inval}}}{\left(\begin{array}{c} T_{\mathrm{total}}\\ T_{\mathrm{inval}} \end{array}\right)}\right)}^{N}$$

where $T_{\mathrm{total}}$ is the number of transactions in the block and $T_{\mathrm{inval}}$ the number of transactions to be validated by each validator node. There are $\left(\begin{array}{c} T_{\mathrm{total}}\\ T_{\mathrm{inval}} \end{array}\right)$ ways to choose a subset of $T_{\mathrm{inval}}$ transactions to be validated.

## 4. Realization of Blockchain Transaction in IIoT

**Figure 4:**
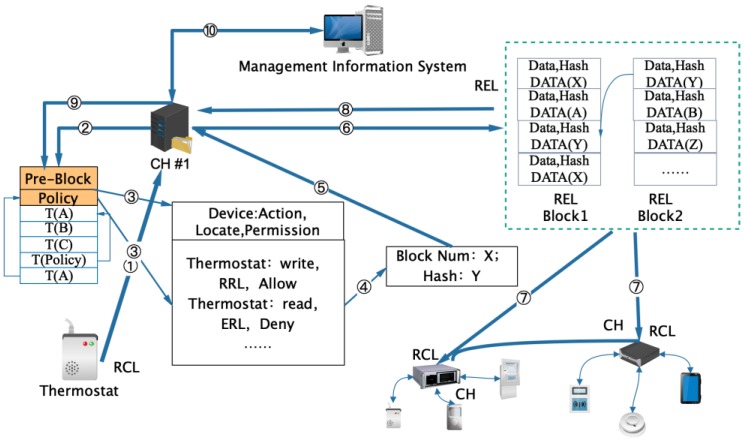
RCL Blockchain generation process for industrial Internet.

Heterogeneous devices of the industrial Internet are managed by various cluster center nodes CH in the local RCL. CH centrally manages an immutable local registration table (LIB), which has a structure similar to blockchain table. All transaction logs are stored in the transaction section of the LIB table for future audits. The asymmetric encryption method is used to encrypt the local transaction. The shared key is established between the two parties, and the lightweight cryptographic hash function is used. Taking the data storage transaction in RCL of layered blockchain as an example, Figure 4 described the process of generation and right confirmation of the acquisition data request block of a device No. 0 (thermostat). The flow chart is as follows:

(1) The local data request device (thermostat) initiates a data exchange request to the control device of the CH1, and the shared key between the two entities is encrypted. At the same time, the requesting device creates LIB ledger locally.

(2) The control device inquires whether the local block right confirmation requesting device has the corresponding permission. This permission table (including the control of read and write permission for temperature control devices in this example) has been written to the blockchain by RCL when creating the original block.

(3) The execution policy part of the block table encrypts and stores all the policy definitions of RCL cluster devices. The execution policy stores all the policies of RCL cluster devices in blocks by a linked list. The current block requests the current operation according to the execution policy stored in the previous block.

(4) Under the control of current operation authority, according to the requirement of reading or writing or storing data, a new block number X is generated. At the same time, the validity of the transaction request is checked according to the corresponding block and hash value, and it is written into the RCL block structure.

(5) The new block data generated by RCL CH1 and the hash Y value are calculated. If two hashes match, the transaction is acceptable, and the transaction results are stored according to the execution policy.

(6) The generated new block X is encrypted with the shared key and then sent to the REL control device, where REL completes the consistency check and updates the REL block.

(7) Hash operation of the new block is carried out and sent back to the blockchain network. According to the distributed right confirmation mechanism, block verification of each cluster center node is completed, and the block information of each node of RCL is updated.

(8) Return the new block number and update RCL local blockchain through the device to complete the transaction process of layered blockchain.

In this process, the CH node that has forwarded the transaction must retain the hash value in the RCL block chain. In addition, the CH of the requester and requested also records the transaction. Other nodes decide whether they are related to the transaction or not, and if there is no correlation, they do not run themselves. After verifying the consistency of the REL layer, the node block information is updated, which reduces the traffic of the whole network and improves processing efficiency of the blockchain.

## 5. Experimental Simulation

### 5.1. Evaluation of the Distributed Trust Algorithm

**Figure 5:**
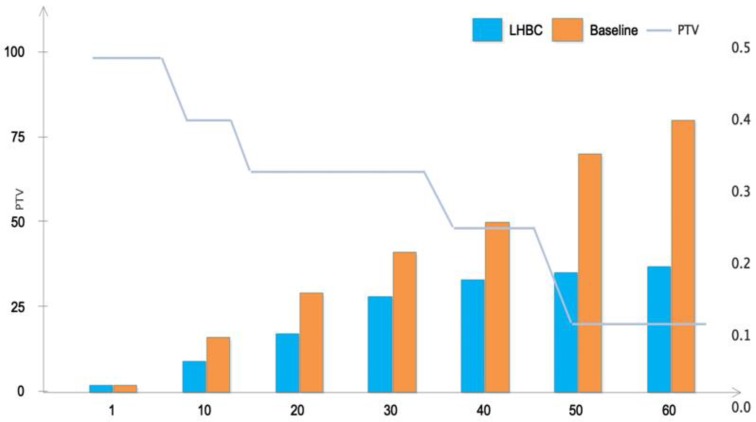
LLBF block verification performance evaluation.

In this section, the performance of the trust mechanism of LLBF blocks is evaluated to test the viability of running blockchain platforms on IIoT devices. We conducted simulations with the default configuration with 20 nodes acting as CHs. To test the viability of running blockchain platforms on IIoT devices we installed and ran different nodes from different platforms on a Raspberry Pi 3 model B with Raspbian Stretch OS and Linux kernel 4.9. In the resource constrained blockchain layer, cluster nodes participated in block generation, verification and consensus in the RCL blockchain network. Because CH nodes were known in the network and had the authority to generate blocks, they did not need to use expensive block excavators to compete for block generation. After receiving all the associated device request transactions, CH verified these transactions and calculated the evidence and device reputation. The CH node waited for its turn to multicast the block to other blockchain nodes for verification. The block generation period time of the gateway can be adjusted according to the sensor data rate and the delay of data collection and block generation. Firstly, a network consisting of 50 nodes was initialized, using default network configuration and node trust right confirmation table (as shown in Table 1). The single simulation lasted 120 s and ran 10 times, and the results were compared with the standard baseline as shown in Figure 5. The left ordinate represents the verification time of each CH block, and the right ordinate represents the Percentage of the Transactions Validated (PTV).

When the LLBF block verification started, the processing time of the two methods was the same, because CH had not yet gained mutual trust. Over time, however, more blocks were generated and verified in RCLBC, and direct trust was established between CH. Compared with baseline, LLBF only needed to verify a small part of the total transactions in the new block, which reduced processing time. In addition, as the number of verification blocks increased, the volume of transactions gradually decreased and CH indirect trust continued to increase. As shown in Dynamic Trust Table 1, once 60 blocks were generated, the trust between CHs reached the highest level, and the number of transactions to be verified and the processing time remained basically unchanged. When the operation reached a stable state, the block verification efficiency of RCLBC improved by 40% compared with the baseline algorithm. 

The proposed block trust mechanism adapted the block validation scheme based on the reputation of the block generating CH node and the number of validator nodes N. The integration of trust management in the block verification mechanism improved the block validation and was managed by the CH reputation module. Based on these observations, we considered an adaptive block validation mechanism, where the PTV decreased with the reputation of the CH block generating node and the number of validator nodes (N). PTV decreased rapidly with the increase of N, which may have reduced the probability of detecting invalid blocks. For smaller validation numbers, increasing N did not reduce PTV sufficiently, and resulted in more transactions being validated than required. The adaptive block verification scheme in this paper can significantly reduce the calculation cost of the block verification process and improve the scalability and delay of the trust architecture.

**Figure 6:**
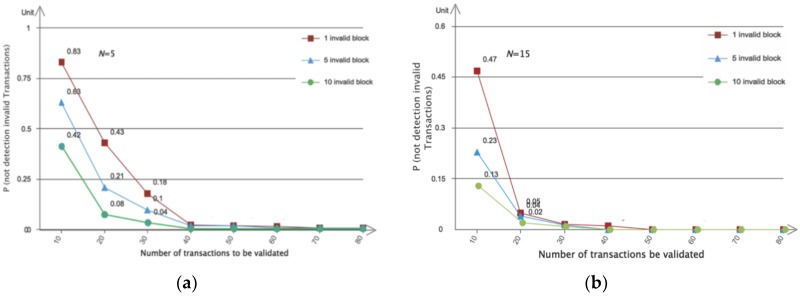
Probability of not detecting invalid transactions in a block. Total = 80 and N = 5, 15 respectively: (**a**) N = 5; (**b**) N = 15.

Figure 6 shows the impact of the number of validators and invalid transactions in a block on the probability that no invalid transactions are detected during block validation (based on Formula 2 in Section 3.4). As the number of validators increased, we could reduce the percentage of transactions that needed to be validated without compromising the performance of invalid transaction detection. When each validator only validated 40% of the transactions, the probability of not detecting an invalid transaction in 80 transactions was less than 0.3%. If there were multiple invalid transactions (when the number of invalid transactions is 5, 10 respectively), the probability of not detecting invalid transactions was significantly reduced.

### 5.2. DHM Performance Analysis

**Figure 7:**
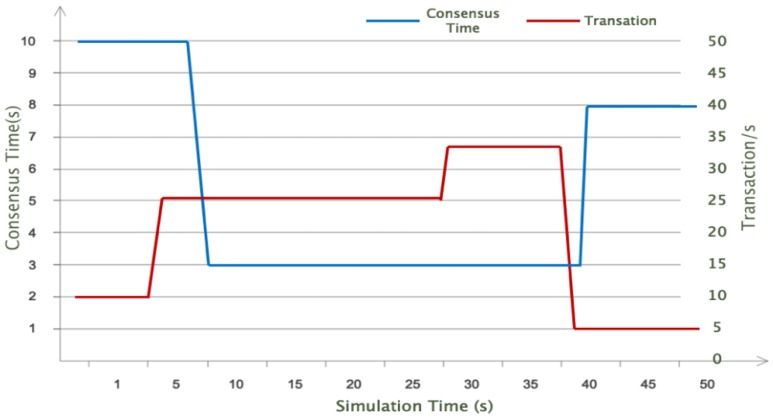
Dynamic regulation of blockchain utilization efficiency.

In order to verify the performance of DHM mechanism when the total load changes dynamically, we simulated the network throughput experiment with 20 CH nodes. Different from the fixed throughput of seven transactions per second of traditional Bitcoin BC, the experiment adjusted the network throughput through different time periods: the experiment adjusted the number of transactions generated in the network at 5, 30 and 40 s, respectively. At the 5th second, the network throughput increased from 2 to 25 per second and remained at 30s. At the 30th second, the load was further increased to 33 transactions per second. Until the 40th second, the load dropped to five transactions per second. The related experimental parameters are shown in Table 2. $\theta_{\min}$ and $\theta_{\max}$ were set to 0.2 and 1 respectively, and the consensus cycle was initially set to 10s. As described in Section 2.3, the DHM algorithm of equation 1 dynamically adjusts the consensus cycle to ensure that the utilization rate of blockchains remains within the effective range. The change of network load during the experiment is shown in Figure 7.

When the first consensus cycle (i.e., 10 s) ended, the network load increased sharply to 25s at the 5th second, and θ was calculated to be 2.17 greater than $\theta_{max}$. The system automatically reduced the utilization ratio of blockchain network, and DHM reset the initial value of θ to 0.6, which was the median of $\theta_{min}$ and $\theta_{max}$. DHM used formula 1 to shorten the consensus cycle to 3 s. Subsequently, the network load remained stable until 30s, so the consensus cycle remained unchanged. After 30s, the network load increased further, and θ was calculated to be 0.78, which was still less than $\theta_{max}$, so the system did not need to take further measures. At the same time, the robustness and validity of initializing θ to the median value of $\theta_{min}$ and $\theta_{max}$ were also shown. At the 40th second, the network load dropped sharply to 5, and the value of θ calculated by the root formula dropped to 0.17 at the same time. Since this value was less than $\theta_{min}$, DHM automatically initialized θ as the median and increased the consensus cycle to 8 s. The process of system change is shown in Figure 7.

### 5.3. Summary

Through the above experiments, we analyze the complexity and network overhead of this algorithm. Experimental analysis shows that the efficiency of block chain structure increases with the network scale. The delay in this paper’s time-congruence algorithm is not affected by the network size, but the delay of the CH node adding a new block to the blockchain. The function of Dynamic Trust Confirmation algorithm is also unaffected by the network size. When the load on the network increases, there are two situations: First, DHM adjusts the consensus cycle, which may affect the delay of consensus. The second is tantamount to adjust the number of CH managing network throughput. Because transactions are broadcast between CHs, the complexity of communication overhead inherits from the underlying peer-to-peer communication protocol, and the complexity of communication depends on the underlying algorithm used in the peer-to-peer network, independent of the block validation itself.

In this paper, the blockchain mechanism separates the data flow from the transaction flow. LLBF brings lower packet overhead and transmission latency. The package overhead only increases as the number of CHs increases. Since packets are routed directly to the requester, end-to-end latency is not subject to the number of CHs. It also can be observed that with time going, CHs generates and verifies more and more blocks, and mutual trust and relationship is established between them. The number of verifying transactions gradually decreases as CHs establishes Trust between each other. Conversely, as the number of validated blocks increases, the number of transactions that need to be validated reduces. Once 60 blocks are produced, trust between the CH reaches its highest level. From here, the number of transactions to verify and the processing time is fixed.

## 6. Conclusions

In this paper, we propose a LLBF for IIoT edge computing application. The corresponding blockchain operation is designed for the edge devices with different resource capabilities. In RCL, resource constrained nodes are subdivided into clusters to maximize the scalability. In order to reduce the asynchronism of block operation in network delay, a time consistency algorithm is designed, which limits the number of different blocks generated in the consensus cycle. To improve the throughput of blockchain, a dynamic trust right confirmation algorithm is designed. Each node accumulates evidence about other nodes based on the validity of the original blocks they generate. A high throughput management mechanism is proposed to determine the blockchain efficiency. Finally, an operation example of locally resource constrained device on data transaction simulation experiment is presented. We have implemented data trust mechanisms in RCL blockchain performance analysis. In the future, we will further explore the blockchain implementation mechanism among the RCL, REL and Cloud-computing layer, so as to expand their application areas, and conduct quantitative research and analysis on their operational efficiency.

## Figures and Tables

**Table 1:** Dynamic trust right confirmation mechanism.

**Absolute Trust**	Number of previously verified blocks	10	20	30	40	50	60
	The ratio that needs to be confirm (%)	90	70	50	40	30	20
**Relative Trust**	The mutual trust rate reached by the central node (%)	20	40	50	60	80	100
	The ratio that needs to be confirm (%)	80	70	60	40	30	10

**Table 2:** Experimental parameters of dynamic high throughput management (DHM) efficiency.

Time(s)	Throughput/s	*CH* Number (*N*)	Consensus Cycle (s)	θ
1 s	2	12	10 s	0.4
5 s	25	12	3 s	0.6
10 s	25	12	3 s	0.6
20 s	25	12	3 s	0.6
30 s	33	12	3 s	0.78
40 s	5	12	8 s	0.6
50 s	5	12	8s	0.6
